# Effects of disease-modifying therapy on peripheral leukocytes in patients with multiple sclerosis

**DOI:** 10.1007/s00415-019-09690-6

**Published:** 2020-02-08

**Authors:** F. Schweitzer, S. Laurent, G. R. Fink, Michael H. Barnett, H. P. Hartung, C. Warnke

**Affiliations:** 1 Department of Neurology, University of Cologne, Faculty of Medicine and University Hospital Cologne, Kerpener Straße 62, 50937 Cologne, Germany; 2 Cognitive Neuroscience, Institute of Neuroscience and Medicine (INM-3), Research Center Jülich, Jülich, Germany; 3 Department of Neurology, Royal Prince Alfred Hospital, and Brain and Mind Centre, University of Sydney, Sydney, Australia; 4 Department of Neurology, Medical Faculty, and Center for Neurology and Neuropsychiatry, LVR Klinikum, Heinrich-Heine-University Düsseldorf, Moorenstr. 5, 40225 Düsseldorf, Germany

**Keywords:** Disease-modifying therapy (DMT), Multiple sclerosis (MS), Lymphopenia

## Abstract

Modern disease-modifying therapies (DMTs) in multiple sclerosis (MS) have variable modes of action and selectively suppress or modulate the immune system. In this review, we summarize the predicted and intended as well as unwanted adverse effects on leukocytes in peripheral blood as a result of treatment with DMTs for MS. We link changes in laboratory tests to the possible therapeutic risks that include secondary autoimmunity, infections, and impaired response to vaccinations. Profound knowledge of the intended effects on leukocyte counts, in particular lymphocytes, explained by the mode of action, and adverse effects which may require additional laboratory and clinical vigilance or even drug discontinuation, is needed when prescribing DMTs to treat patients with MS.

## Introduction

Multiple sclerosis (MS) is the most common inflammatory disease of the central nervous system (CNS), presenting in the majority of cases with an initially relapsing–remitting (RRMS) disease course that later converts into a secondary progressive (SPMS) form. A minority of patients will display a primary progressive (PPMS) variant from disease onset, often characterized by a progressive gait disorder in the absence of relapses [1, 2]. Strong evidence indicates an essential role of auto-reactive immune cells in the pathogenesis of MS, encompassing CD4+ and CD8+ T cells as well as B cells [3–5].

Several oral and infusible disease-modifying therapies (DMTs) have been approved for the treatment of RRMS. The approval was based on efficacy in phase III clinical trials, with beneficial effects on clinical measures (relapse rate and to a variable extent disability progression) and on magnetic resonance imaging metrics of inflammatory activity and lesion load (new or enlarging T2-lesions, gadolinium-enhancing lesions, and to a variable extent also brain atrophy) [6]. Recently, with the approval of ocrelizumab for early PPMS and siponimod (registered in the USA, positive Committee for Human Medicinal Products (CHMP) opinion in the EU) for active SPMS [7], the first treatment options for progressive forms of MS became available (Table 1).

**Table 1:** DMTs for the treatment of MS*

Treatment (alphabetic order)	Indication	Route of administration	Dosing/interval
Alemtuzumab (Lemtrada^®^)	RRMS	i.v.	5 × 12 mg (1st year) and 3 × 12 mg (2nd year)/short pulsed treatment periods
Cladribine (Mavenclad^®^)	RRMS	p.o.	3.5 mg/kg (1.75 mg/kg per year) body weight over 2 years/short pulsed treatment periods
Dimethyl fumarate (Tecfidera^®^)	RRMS	p.o.	Maintenance dose 240 mg bid
Fingolimod (Gilenya^®^)	RRMS	p.o.	0.5 mg/day (in adults)
Natalizumab (Tysabri^®^)	RRMS	i.v.	300 mg/month, *off-label*: extended interval dosing (EID) [14]: 300 mg/every 6–8 weeks
Ocrelizumab (Ocrevus^®^)	RRMS, PPMS	i.v.	Induction 300 mg at day 0 and 14; maintenance dose 600 mg/every 6 months
Rituximab (*off-label*) (MabThera^®^, Truxima^®^, Rixathon^®^)	RRMS	i.v.	Induction and maintenance dose variable, mostly 500 mg/every 6 months
Siponimod (Mayzent^®^)	RRMS (FDA), active SPMS (CHMP vote [7])	p.o.	Maintenance dose dependent on the CYP2C9 genotype: 2mg/day, or 1mg/day, or contraindicated
Teriflunomide (Aubagio^®^)	RRMS	p.o.	14 mg/day

*Injectables (interferon beta and glatiramer acetate) without immunosuppressive potential not listed

DMTs selectively suppress or modulate the immune system either by depleting particular (sub-)populations of leukocytes, or by targeting lymphocytes through interference with activation, proliferation, cytokine production, or trafficking across the blood–brain barrier [3, 8]. As a result of a distinct mode of actions, changes of the peripheral lymphocyte counts are treatment specific and may require different monitoring and management. As such, unexpected adverse effects on lymphocyte counts differ for the individual DMTs. Some of these laboratory changes have been associated with an increased risk of infections or other adverse clinical events [9–13]. The total number of lymphocytes (extent of lymphopenia), distinct changes in the overall leukocyte subpopulations, as well as the timing and sequence of immune reconstitution following cell depletion, have been related to possible therapeutic risks that include secondary autoimmunity, infections, and impaired response to vaccinations.

## DMT modes of action and lymphopenia

Except for natalizumab (and the injectables interferon beta and glatiramer acetate, not discussed in this review), all recent DMTs can cause variable degrees of lymphopenia, which can be classified—in accordance with the common terminology criteria for adverse events [15]—by the absolute lymphocyte count (ALC) per cubic millimeter (mm^3^) of blood as follows:

- *Grade 1* (*mild lymphopenia*) ALC < lower limit of normal to 800/mm^3^
- *Grade 2* (*moderate lymphopenia*) ALC < 800–500/mm^3^
- *Grade 3* (*severe lymphopenia*) ALC < 500–200/mm^3^
- *Grade 4* < 200/mm^3^

However, when evaluating the clinical relevance and severity of lymphopenia, it should be recalled that circulating lymphocytes make up only approximately 2% of the body’s total lymphocyte population and thus provide only limited information on the overall immune status of a patient [16, 17]. Also, lymphocyte counts are highly variable both intra- and inter-individually, and the “normal” range thus needs to be redefined for each of the different DMTs after treatment initiation. Currently, known effects of DMTs on the immune system, in particular on lymphocyte counts, are summarized in Table 2 and discussed further in the following section of this review.

**Table 2:** Effect of disease-modifying therapies in RRMS on circulating lymphocytes

Treatment	Degree of associated lymphopenia	Effect on circulating lymphocytes (reduction from baseline)	Lymphocyte recovery to LLN/baseline
Alemtuzumab (Lemtrada^®^)	Grade 3 and 4: 99.9% [18]	1 month post-treatment initiation:	Total lymphocytes: 6–12 months in 40 and 80% of patients (after each treatment cycle) CD4+ T cells: 12 months: 10–20% patients CD8+ T cells: similar to total lymphocyte count CD19+ B cells: 6 months in ≥ 85% [19]
		Depletion of circulating lymphocyte to a mean of 250/mm^3^ and 320/mm^3^ blood during treatment courses 1 and 2 [18]	
Cladribine (Mavenclad^®^)	Phase III trial (CLARITY): Grade 3: 25.6% Grade 4: 0.7% [27]	Phase III trial (CLARITY): 4 months post-treatment initiation in year 1:	Phase III trial (CLARITY): Recovery of lymphocytes at the end of each treatment year: 86% [27]
		Total lymphocyte counts: 42%	
		2 months post-treatment initiation in year 2:	
		Total lymphocyte counts: 58% [27]	
Dimethyl fumarate (Tecfidera^®^)	1 Phase IIb and 3 Phase III trial (DEFINE, CONFIRM, ENDORSE): Grade 1: 9% Grade 2: 21% Grade 3: 7% Grade 4: < 1% [35]	12 months post-treatment initiation:	4 weeks: increased, but did not return to baseline [39]
		Total lymphocyte counts: 30% [64]	
Fingolimod (Gilenya^®^)	Grade 4: 18% [43]	Dose-dependent reduction:	1–2 months [43]
		Total lymphocytes: 70–80% [43]	
Natalizumab (Tysabri^®^)		increases in peripheral lymphocytes [14]	16 weeks [14]
Ocrelizumab (Ocrevus^®^)	RRMS: < LLN: 20.7% PPMS: < LLN: 26.3% Grades 1 and 2: majority Grade 3: 1% [58]	2 weeks of treatment (first time point of assessment):	2.5 years: 90% of patients had recovered CD19+ B cells (median: 72 weeks) [58]
		Depletion of CD19+ B cells	
		No alterations in T cells [58]	
Rituximab (MabThera^®^, Truxima^®^, Rixathon^®^)		Phase II trial (OLYMPUS): 2 weeks of treatment:	Phase II trial (OLYMPUS): 48 weeks: 35% of patients had recovered CD19+ B cells [59]
		CD19+ B cells: 95%	
		No alterations in CD3+ T cells [59]	
Siponimod (Mayzent^®^)	Phase III trial (EXPAND): Grade 4: 1% [47]	Dose-dependent reduction:	10 days: 90% of patients [47]
		Total lymphocytes: 70–80% [47]	
Teriflunomide (Aubagio^®^)	Phase III trials (TEMSO, TOWER, TENERE, TOPIC): Grade 1: 7.3% Grade 2: 2.2% No grade 3 or 4 lymphopenia was reported [62]	6 weeks of treatment:	Recovery from grade 1 lymphopenia: 10.6 weeks Recovery from grade 2 lymphopenia: 16.6 weeks [63]
		Decrease in white blood cell count: 15% (mainly neutrophils and lymphocytes) [62]	

### Alemtuzumab

Treatment with alemtuzumab, an anti-CD52 monoclonal antibody, targeting T and B cells, monocytes, dendritic cells, and thymocytes, results in a substantial and sustained depletion of circulating lymphocytes (grades 3 and 4 lymphopenia: 99.9% of patients) [18]. Immune reconstitution varies for lymphocyte subpopulations. After each treatment cycle, approximately 40% and 80% of patients in phase III clinical studies reached ALC at the lower limit of normal (LLN) by 6 and 12 months, respectively. While the repopulation of B cell counts (CD19+) occurred early (LLN in ≥ 85% by 6 months), CD8+ T cells showed similar repopulation kinetics as compared with the ALC, while CD4+ T cells displayed a longer-lasting depletion (only 10–20% of patients with CD4+ cells above the LLN by month 12) [19–21]. In addition to the reduction in lymphocytes, in 16% of the alemtuzumab-treated patients mild neutropenia could be observed, whereas severe neutropenia occurred in 0.6% [22].

Differences between B cell depletion in peripheral blood and primary and secondary lymphoid organs have been observed in mouse models, as most lymphocytes were depleted in the circulation, but could still be detected in significant numbers in the spleen, lymph nodes, bone marrow, and thymus [23]. This might explain the rapid B cell repopulation after alemtuzumab treatment in MS patients, assuming that also in humans, B cells are at least partly preserved in the lymphoid organs.

The comparably rapid repopulation of B cells, in the absence of CD4+ T regulatory cells and CD8+ T suppressor cells, has been implicated in the development of secondary autoimmune diseases (such as thyroid disorders, immune thrombocytopenia, and autoimmune nephropathies), which are a common side effect observed in patients treated with alemtuzumab [24]. Nonetheless, following the treatment with alemtuzumab, cases of T-cell-driven secondary autoimmunity, including vitiligo and alopecia, have also been described [25, 26].

### Cladribine

Cladribine (2-chloro-20-deoxyadenosine; CdA) is a synthetic deoxyadenosine analog that is preferentially activated by intracellular phosphorylation in specific cell types, resulting in a targeted reduction of circulating lymphocytes. Lymphocyte reductions following treatment with cladribine develop gradually, due to depletion of cells via an apoptotic mechanism, in contrast to the very rapid reductions seen after treatment with monoclonal antibodies, which have a cytolytic mode of action [11].

The pivotal CLARITY phase III study showed that 4 months after treatment initiation in year one, total lymphocyte counts decreased by 42%, while 2 months after the second cycle in year 2, lymphocytes decreased by 58%. Lymphocytes recovered in 86% of the patients at the end of each treatment year. Grade 3 lymphopenia was experienced in approx. 26% of patients and grade 4 in 0.7% [27]. The absolute numbers of monocytes and neutrophils remained unchanged following cladribine treatment [28–32].

### Dimethyl fumarate (DMF), monomethyl fumarate (MMF)

To date, the modes of action of DMF and its active metabolite MMF have not been fully elucidated. DMF and MMF modulate the activity of signaling proteins, including the transcription factors nuclear factor erythroid 2-related factor 2 (Nrf2) and nuclear factor kappa-light-chain-enhancer of activated B cells (NFkB), believed to prompt generation of cytoprotective factors and the stimulation of anti-inflammatory and inflammatory signals [33]. However, DMF might also elicit an initial and short-lived oxidative stress by binding and sequestering the intracellular antioxidant glutathione before activating the antioxidant response and subsequent protection against reactive oxygen species [34].

Combined data of one phase IIb and the DEFINE, CONFIRM, and ENDORSE phase III clinical trials showed grade 1 lymphopenia in 9% of patients, grade 2 in 21%, grade 3 in 7%, and grade 4 in < 1% [35]. Older patients (> 55 years of age), patients with lower baseline lymphocyte counts, and patients with recent natalizumab exposure appeared to be at a higher risk of lymphopenia [36–38]. Four weeks post-treatment, mean lymphocyte counts increased but did not return to baseline [39].

### Sphingosine-1-phosphate receptor modulators: Fingolimod, Siponimod

Fingolimod is the first-in-class sphingosine-1-phosphate receptor (S1PR) modulator and preferentially inhibits CCR7+ lymphocytes egress from secondary lymphoid organs, resulting in a profound diminution of naive and central memory T cells and memory B cells in the periphery [40–42]. A dose-dependent decrease in total peripheral lymphocytes to 20–30% of baseline (reduction by 70–80%) can be observed. Lymphocytes generally return to the normal range at about 1–2 months after treatment discontinuation [43]. According to EU guidelines, treatment should be stopped when the ALC drops below 200/mm^3^ and until the ALC rises above 600/mm^3^ [44].

Siponimod is a second-generation, more selective S1PR1 and S1PR5 modulator and synthetic derivative of fingolimod that has been approved in the USA for treatment of CIS, RRMS, and early SPMS and recently received a positive opinion by the Committee for Human Medicinal Products (CHMP) for active SPMS in the EU [45] and for SPMS by the Australian TGA [46]. Siponimod leads to a dose-dependent reduction of peripheral lymphocytes by 70–80%, with a recovery to the normal range within 10 days in 90% of the patients after treatment discontinuation. However, lymphocyte recovery can take up to 3–4 weeks in some patients. In the pivotal phase III EXPAND study, grade 4 lymphopenia was observed in 1% of patients [47, 48].

### Natalizumab

Natalizumab, an anti-α4 integrin monoclonal antibody, prevents lymphocyte migration through the blood–brain barrier into the CNS [49]. In contrast to the other immunosuppressive DMTs discussed in this review that associate with lymphopenia, natalizumab treatment leads to a considerable increase in peripheral blood of CD4+ and CD8+ T cells, CD19+ B cells, and NK cells in the peripheral blood, due to elevated release or impaired homing of lymphocytes, including the CD34+ progenitor cells subpopulation, from or to the bone marrow and secondary lymphoid tissues, respectively [50, 51]. Compartmentalized effects are noted, with the total number of lymphocytes in the cerebrospinal fluid (CSF) being dramatically reduced and a diminution of the CD4+/CD8+ ratio. After cessation of treatment, circulating lymphocytes return to baseline levels usually within 16 weeks, and the CD4+/CD8+ ratio within the CSF normalizes within 6 months [52, 53].

### Ocrelizumab and rituximab

Ocrelizumab and rituximab are both anti-CD20 antibodies, which, however, bind different epitopes on the CD20 antigen expressed on the surface of cells during distinct stages of the lymphocyte lineage. While rituximab is a chimeric antibody, composed of mouse and human parts acting primarily via complement-dependent cytotoxicity (CDC), ocrelizumab is a humanized anti-CD20 monoclonal antibody with a proposed higher capacity for direct, antibody-dependent cellular cytotoxicity (ADCC), and to a lesser extent CDC [54, 55].

Ocrelizumab has been approved for the treatment of both RRMS and early PPMS [56, 57]. Following the first infusion, B cells were largely depleted after 2 weeks and remained depleted throughout treatment. 20.7% of RRMS and 26.3% of PPMS patients showed a drop in total lymphocyte counts, with the majority showing mild lymphopenia (> 500 cells/mm^3^) and only 1% severe grade 3 lymphopenia (200–500 cells/mm^3^). No patient was reported with a lymphocyte count of fewer than 200 cells/mm^3^. 13% of PPMS patients had only a small decrease in neutrophils reported as mild neutropenia, whereas 1% had severe neutropenia [58].

Rituximab causes a selective, transient long-lasting depletion of CD20+ B cells. In the phase II OLYMPUS clinical trial in primary progressive MS, a reduction of CD19+ B cells by 95% was detected 2 weeks after treatment initiation. B cells recovered in 35% of patients within 48 weeks after treatment discontinuation [59]. In the HERMES trial, which evaluated rituximab as a treatment in adults with RRMS, the treatment was associated with a rapid and nearly complete (> 95%) depletion of CD19+ B cells from 2 weeks after treatment initiation until 24 weeks. By week 48, B cells recovered to baseline in 30.7% of patients [60].

### Teriflunomide

Teriflunomide selectively inhibits the mitochondrial enzyme dihydroorotate dehydrogenase, thereby preferentially targeting proliferating recently antigen-activated lymphocytes. As a result, the proliferation and function of recently activated T and B cells (thought to contribute to the inflammatory processes in MS) are reduced, while the resting cells of the adaptive immune system are largely spared [61]. A reduction of about 15% from the baseline level in white blood cells can be detected within the first 6 weeks of treatment [62]. In a pooled analysis of the clinical studies, including TEMSO, TOWER, TOPIC, and TENERE phase III trials, mild lymphopenia was infrequent in both the core and extension studies (grade 1: 7.3%; grade 2: 2.2%), and no severe lymphopenia was reported. In the case of grade 1 lymphopenia, lymphocyte recovery time was 10.6 weeks, and in the case of grade 2, 16.6 weeks [63].

## Specific risks of infections that are associated with changes in leukocyte counts

MS patients undergoing treatment with immunosuppressive DMTs will have an increased overall risk for mild and severe infections, including rare but severe opportunistic infections, due to alterations of protective immune responses [65, 66]. These infections can be caused by the reactivation of a latent pathogen, the worsening of previously asymptomatic chronic infections, and a higher susceptibility to new infections. Several DMTs have been associated with a significant increase in the risk for mostly mild or moderate infections, such as nasopharyngitis, upper respiratory tract infections, sinusitis, oral herpes, influenza, bronchitis, and urinary tract infections, none of which require specific preventive measures. More serious opportunistic infections associated with DMT will be discussed in the following section.

### *Cryptococcus neoformans* infection

Cases of cryptococcal meningitis (i.e., a fungal infection) have been described in patients undergoing fingolimod treatment for 2–3 years [44]. Although a causal link between fingolimod-induced CD4+ T cell lymphopenia and cryptococcus infection has not been proven, decreased CD4+ T cell counts might be a risk factor for acute cryptococcal infection or a reactivation of a latent infection [67].

### Hepatitis B virus (HBV) infection

MS patients with a known latent HBV infection who receive certain immunosuppressive therapies are at risk for viral reactivation. Treatment with anti-CD20 monoclonal antibodies, such as ocrelizumab and rituximab, comes with an increased risk of HBV-associated hepatitis and liver failure [68]. Prophylactic treatment with antiviral drugs such as tenofovir/entecavir is recommended, before, during, and up to 12 months after the last dose in this patient subgroup, while patients with active HBV infection should not receive B cell depleting therapy [69].

### Herpes virus infection

Immunosuppressive therapy, particularly therapy affecting cellular immunity, elevates the risk of reactivation of latent herpes simplex virus (HSV) 1 and 2 and varicella zoster virus (VZV) infection. In particular, alemtuzumab administration and cladribine administration were associated with increased rates of HSV and VZV infections, in some cases requiring hospitalization due to generalized infections. As a consequence, guidelines include prophylactic therapy with acyclovir (200 mg twice daily p.o.) after alemtuzumab initiation (in the first month of the therapy) and cladribine (if lymphocytes fall below 200/mm^3^) [69]. Increased risk of herpes viral infections has also been reported for DMTs such as fingolimod, natalizumab, dimethyl fumarate, and ocrelizumab [70], arguing for close clinical vigilance and effective vaccination strategies including VZV (see below).

### Listeria monocytogenes infection

Listeria monocytogenes is a facultative intracellular Gram-positive bacillus, typically ingested via contaminated food, such as dairy products, raw fish, and meat. Severe symptoms are rare in immunocompetent persons, whereas people with defective cellular immunity may develop septicemia, meningitis or encephalitis, with a mortality rate ranging from 20 to 40%. Immunosuppression (of various causes) is a generally well-recognized predisposing risk factor. Listeriosis resulting in acute meningitis has been described in the context of treatment with DMF and alemtuzumab for MS [71–74]. A ‘Listeria diet’ is recommended prior to and after commencing treatment with alemtuzumab.

### Progressive multifocal leukoencephalopathy (PML)

JC polyomavirus (JCPyV), the causative agent of PML, is common in the general population with a reported prevalence of 30–70% and a continuous rise with age [75, 76]. Drug-related PML has been described with CD4+ and CD8+ T cell deficiencies [77, 78].

As of March 2019, more than 814 cases of PML have been reported in association with natalizumab therapy. As of May 2019, 26 fingolimod-associated PML cases [79, 80], and, as of June 2019, 7 cases associated with dimethyl fumarate (Tecfidera^®^) for MS have been identified that were not biased by prior natalizumab therapy (Novartis and Biogen, data on file). Recently, a single case of a 78-year-old PPMS patient treated with ocrelizumab without prior immunotherapy was reported to develop and succumb to PML (Roche, data on file). Immunosenescence may contribute to a heightened risk of PML also for patients treated with DMF and fingolimod [81]. Additional cases of PML occurred during ocrelizumab therapy in patients switched from natalizumab or fingolimod (so-called carryover cases) (Roche and Novartis, data on file).

### Tuberculosis (TB)

Testing for TB is recommended in particular for patients from countries with high TB incidence, as immunosuppressive medication can result in reactivation of latent bacteria, previously under the control of the immune system [82]. DMTs targeting T-cell-mediated immunity are most likely associated with TB reactivation, whereas those targeting B cells, such as the anti-CD20 monoclonal antibodies ocrelizumab and rituximab, are expected to have a smaller effect [65]. TB screening, e.g., interferon-γ release assay (IGRA), is recommended in particular before alemtuzumab and cladribine therapy, or if risk factors are present prior to therapy with teriflunomide, fingolimod, natalizumab, dimethyl fumarate, or ocrelizumab [83].

## Vaccination responses

Given the heightened risk of infections in patients treated with DMTs, protection against preventable diseases is particularly relevant. In an ideal situation, comprehensive immunization should be completed 6 weeks before the initiation of treatment with DMTs [19, 58, 84].

In general, inactivated, subunit, recombinant, polysaccharide, and conjugate vaccines such as influenza, pneumococcus, hepatitis A and B, tetanus, diphtheria, pertussis, human papillomavirus (HPV), and herpes zoster (“Shingrix”) can be administered to persons with autoimmune disease or chronic inflammatory disease at any given time point, even while on immunosuppressive therapy. Cumulative evidence suggests that the benefits generally outweigh possible risks [85, 86]. Nonetheless, patient-specific factors need to be considered: Individuals who are experiencing a severe relapse should postpone vaccination until at least 4–6 weeks after the onset of the relapse, and the success of immunization can be reduced during DMT, thus requiring serological assessments.

A clinical study of 71 patients with RRMS and DMF therapy for at least 6 months showed that patients were able to mount sufficient responses to tetanus (recall antigen) and a conjugated meningococcal C polysaccharide vaccine (neoantigen) [64]. Likewise, natalizumab treatment of RRMS patients did not influence the response to tetanus (recall antigen) significantly. In contrast, natalizumab slightly reduced the response to a neoantigen (keyhole limpet hemocyanin, KLH) in a cohort of 60 patients [14]. Ocrelizumab-treated RRMS patients were able to mount responses following vaccinations, although the response rates decreased. This has been studied for tetanus, 23-valent pneumococcal polysaccharide with or without a booster vaccine, KLH, and influenza [58]. In a study of relapsed low-grade non-Hodgkin’s lymphoma who received rituximab, the patients exhibited a lower rate of response to vaccination with tetanus and KLH neoantigen [87]. Two clinical studies have shown that vaccinations to inactivated neoantigen (first vaccination) or recall antigen (re-exposure) were safe and effective during teriflunomide treatment [62].

Immunization with live but attenuated vaccines (MMR, VZV live vaccine, yellow fever), in contrast, should be completed at least 4 weeks prior to treatment initiation, or 6 weeks before depleting agents such as alemtuzumab, ocrelizumab, or cladribine. These vaccinations are contraindicated during the treatment with immunosuppressive DMTs, as the resulting immunosuppression could result in an active infection [19, 58, 88]. Exceptions should only be made in justified individual cases after thorough risk–benefit assessment.

## Alternative dosing strategies

As outlined above, the mode of action for the different DMTs used in MS is variable, resulting in differences in quantitative (extent of lymphopenia) and qualitative (cellular composition) lymphocyte counts. While lymphocyte counts between 200/mm^3^ and 500/mm^3^ (grade 3 lymphopenia) constitute a risk factor for opportunistic infections such as PML in patients treated with DMF, this may not be the case for other treatments such as fingolimod (no association between the grade of lymphopenia and the risk of PML reported so far). In individuals treated with fingolimod, even ALCs below 200/mm^3^ may be tolerated according to US and Swiss treatment guidelines, as no apparent link to an elevated risk of infection could thus far be established. Currently, due to rigorous adherence to treatment regimens that were tested in pivotal phase III clinical trials, MS therapeutics, except cladribine, are administered at an approved standard dose and at approved intervals, regardless of individual body weight, duration of therapy, or other factors that might influence pharmacokinetic parameters, such as drug plasma levels or receptor saturation. While with the use of DMF in psoriasis (Fumaderm^®^), a dose reduction by 50% is recommended by the EMA if the lymphocytes drop below a certain threshold (700/mm^3^) [89]. This is not suggested when using DMF in MS. Similarly, with the use of fingolimod in MS, if lymphocyte counts drop below the EMA threshold of 200/mm^3^, treatment suspension is recommended rather than dose reduction. A reduced dose is indicated for children at lower body weight [44], but not for adults.

Recently, the natalizumab product information has been updated [14], to include data suggestive of a lower risk of PML in patients treated with extended interval dosing (EID) [90]. However, it remains to be demonstrated that EID of natalizumab yields the same degree of efficacy if close clinical and MRI monitoring is performed [91]. A prospective study (ClinicalTrials.gov Identifier: NCT03689972) that assesses the efficacy of 6-week intervals of natalizumab completed recruitment, and the estimated study completion date is September 30, 2021 [92].

Overall, these examples indicate that formal drug labeling and clinical practice may conflict, and individualized shared treatment decisions may be required in some patients that deviate from the exact protocol of phase III clinical trials. This seems particularly relevant for long-term therapy. These considerations may also be applicable for B-cell-depleting agents such as ocrelizumab, ofatumumab, and rituximab, as cumulative suppressive effects on IgG and IgM levels may occur [58].

## Conclusion

DMTs can alter lymphocyte counts, lymphocyte subset distribution, and their trafficking from lymph nodes into the periphery and the CNS. Screening for latent infection as well as comprehensive vaccination should be completed prior to the initiation of treatment using DMTs to avoid severe opportunistic infections, of which some may be linked to lymphopenia. Monitoring of lymphocyte counts should be performed as recommended, and individualized action may be required if lymphocyte counts drop below treatment-specific thresholds (Table 3).

**Table 3:** Risk of lymphopenia, threshold, and possible interventions*

Treatment	Lymphocyte count measurement intervals	Threshold	Intervention
Alemtuzumab (Lemtrada^®^)	Monthly	No lymphocyte threshold (critical are: thrombocytopenia neutropenia, hemolytic anemia, and pancytopenia)	Acyclovir prophylaxis If neutropenia, hemolytic anemia, thrombocytopenia, or pancytopenia occurs, immediate hematological consultation is recommended
Cladribine (Mavenclad^®^)	Min. every 2–3 months	< 200/mm^3^	Acyclovir prophylaxis
Dimethyl fumarate (Tecifidera^®^)	3-Monthly	< 800 (500)/mm^3^	Repeat testing, consider alternative therapy in particular if lymphopenia persists for > 6 months
Fingolimod (Gilenya^®^)	At week 2 and 4 of treatment start; then 3-monthly	< 200/mm^3^	Repeat testing, if confirmed, treatment suspension
Natalizumab (Tysabri^®^)	6-Monthly	–	Lymphopenia not expected, consider hematological consultation if observed
Ocrelizumab (Ocrevus^®^)	3-Monthly	–	Severe lymphopenia not expected, consider hematological consultation if observed; monitor IgG and IgM levels in addition, in particular if frequency of infections increase/severe infections occur
Rituximab (MabThera^®^, Truxima^®^, Rixathon^®^)	3-Monthly	–	See ocrelizumab
Siponimod (Mayzent^®^)	At week 2 and 4 of treatment start; then 3-monthly (adapted from fingolimod, no specific recommendations in the FDA product information [47]	< 200/mm^3^ (in analogy to fingolimod, no specific recommendations in the FDA product information [47]	Repeat testing, if confirmed, treatment suspension (no specific recommendations in the FDA product information [47]
Teriflunomide (Aubagio^®^)	2-Monthly within the first 6 months, then 3-monthly	< 200/mm^3^	Treatment suspension

*According to EMA product information and Qualitätshandbuch MS/NMOSD 2019 [69]
